# Improving adherence to medical regimens for juvenile rheumatoid arthritis

**DOI:** 10.1186/1546-0096-5-10

**Published:** 2007-05-18

**Authors:** Michael A Rapoff, Carol B Lindsley

**Affiliations:** 1 Department of Pediatrics, University of Kansas Medical Center, Kansas City, Kansas, USA

## Abstract

Poor adherence to medical regimens can compromise the efficacy of treatments for children and adolescents with juvenile rheumatoid arthritis (JRA). The purpose of this review is to describe medical regimens for the treatment of JRA and the rates of adherence to these regimens. We also summarize and critically the few research studies aimed at improving adherence to regimens for JRA. Finally, we summarize strategies for enhancing adherence in clinical practice.

## Background

Children and adolescents with juvenile rheumatoid arthritis (JRA) are often asked to adhere consistently and over a long period of time to a variety of medical regimens, most notably, medications and therapeutic exercises. We have chosen to use the term JRA because almost all of the studies in this area have been done with children meeting the American College of Rheumatology criteria for JRA [1]. Regimens for JRA may have delayed beneficial effects and in the short term may cause unwanted side-effects such as gastrointestinal irritation and pain. This constellation of factors associated with treatments for JRA (i.e., the need for consistent adherence over a long period of time, delayed beneficial effects, and negative side-effects) are predictive of greater adherence problems to medical regimens in pediatric chronic disease [2]. This purpose of this review will be to 1) describe current regimens, define adherence, and review the prevalence of nonadherence to regimens for JRA; 2) summarize and critically evaluate research on improving adherence to regimens for JRA; and 3) review strategies for enhancing adherence in clinical practice.

### Medical regimens for JRA

Pediatric rheumatic disease encompasses chronic multi-system disorders that involve acute and chronic tissue inflammation of the musculoskeletal system, blood vessels, and skin. JRA is the most common form of chronic arthritis and a major cause of both short and long-term disability. Patients are often required to adhere to complex medical regimens and cope with pain and psychosocial aspects of their disease. The etiology of JRA is not known but genetic and environmental factors likely are important. The laboratory tests are rarely definitive, the disease evolves over time and the treatment response is often not predictable. Even with good disease control, symptoms may fluctuate with other factors, such as intercurrent infection and weather changes.

Synovitis is the hallmark of the disease and there are three basic subtypes, defined by the first 6 months of disease. Systemic disease represents 10–15% of patients. These patients have high fever, characteristic rash and usually polyarticular arthritis, and may have organomegaly, serositis and risk for macrophage activation syndrome. Those children without major systemic symptoms are divided into polyarticular (≥ 5 joints) and pauciarticular (≤ 4 joints) subtypes. Those children with polyarticular disease usually have symmetric, small joint involvement, particularly hands, wrists and hips. They often have low grade symptoms or signs such as fatigue and anemia and may be positive for rheumatoid factor, a marker for more aggressive disease. Children with pauciarticular (oligoarticular) disease are usually young, have predominantly large joint involvement and a high risk for uveitis, anterior chamber eye inflammation.

The general approach to treatment is a stepwise approach, usually starting with non-steroidal anti-inflammatory agents and aggressively adding additional drugs depending on therapeutic response. Other aspects of treatment may need to address growth abnormalities (including nutrition problems), vision, exercise needs-therapeutic and general, school and social function, and psychosocial and emotional health.

### Defining adherence

"Adherence" is now the preferred term in the literature, replacing the term "compliance". The term adherence better reflects a more active role for patients in consenting to and following prescribed treatments [2,3]. The World Health Organization defines adherence as "the extent to which a person's behavior – taking medication, following a diet, and/or executing lifestyle changes, corresponds with agreed recommendations from a health care provider" [4]. This definition acknowledges that regimens for chronic conditions involve multiple behavioral components, with varying levels of adherence to each component, and that agreement to follow regimens has been secured from the patient. In pediatric rheumatology, agreement to follow prescribed regimens needs to be obtained from caretakers as well as patients.

Another potentially useful distinction in the adherence literature is between inadvertent (non-volitional) and volitional (or intentional) nonadherence [5]. Inadvertent nonadherence may involve patients forgetting to take a medication dose or being away from home without access to medications. Whereas, volitional nonadherence may involve a reasoned and purposeful decision by patients to omit a medication dose, because they are asymptomatic, taking medications that interferes with their lifestyle, or are being defiant. In the case of inadvertent nonadherence, one could help patients problem solve about how to keep track of medication doses (such as using a pill reminder case) and to have medications with them when away from home. In the case of volitional nonadherence, one would need to negotiate with patients and their families to obtain agreement on what they would be willing to do to treat their disease without compromising their quality of life.

### Prevalence of nonadherence to regimens for JRA

There are few studies that have specifically addressed adherence to regimens for JRA. Two retrospective studies by Litt and her colleagues examined adherence to salicylate medications in the treatment of JRA. In the first study [6] adherence among 82 patients with JRA was assessed using serum salicylate assays, with patients classified as adherent if their mean salicylate levels over a 19-month period were above 20 mg/dl. Some 55% of adolescents (n = 38) and 55% of children (n = 44) were found to be adherent, In the second study [7], adherence among 38 adolescents with JRA was assessed using serum salicylate assays obtained over a 12-month period, with patients again classified as adherent if their salicylate levels were greater than 20 mg/dl. Again, 55% of adolescents were adherent.

In three separate within-subject design studies involving five patients with JRA (ages 3 to 14 years), who were suspected of having adherence problems by their pediatric rheumatologist, our research group assessed baseline adherence with salicylates and other medications, including naproxen, penicillamine, prednisone, and tolmetin sodium [8-10]. Adherence was assessed by parental observations or pill counts with independent interobserver reliability assessments conducted by an investigator in the patients' home or in the clinic (average interobserver agreement exceeded 90%). Mean baseline adherence levels with these medications among the five patients ranged 38% to 59%.

A recent study was unique in that it assessed adherence to nonsteroidal antiinflammatory drugs (NSAIDs) among 48 newly-diagnosed children with JRA (mean age = 8.6 years) over 28 consecutive days using an electronic monitoring device [11]. For each participant, each day was defined as being either "full adherence" (all prescribed doses taken on time ± 2-hour forgiveness interval), "partial adherence" (some but not all taken on time), and "no adherence" (no doses taken at all). A "drug holiday" was defined as two or more consecutive days with no doses taken preceded and followed by at least one day with at least partial adherence. Using median levels, patients showed full adherence on 70% of the monitored days, partial adherence on 14%, and no adherence on 7%. There was considerable variability across patients, with full adherence ranging from zero to 100% of the days. Seventy-nine percent of patients took no drug holidays, 13% took one, and the remaining 8% between two and four. Using an 80% adherence cut point, 25 patients (52%) were classified as adherent and 23 (48%) as nonadherent.

Patients with JRA are also asked to adhere to regimens other than medications, such as therapeutic exercise and wearing joint splints. Three studies have assessed parental and patient perceptions of adherence problems with these types of regimens as well as medications. In the first study [12], an adherence questionnaire was administered to 37 parents of children with JRA. The children were prescribed medications and range-of-motion exercises, splints, or both. Parents rated the degree of difficulty they have in motivating their children to adhere to the different types of regimens and noted any negative reactions their children had to the regimens. Parents reported more problems with prescribed exercises as compared with medications or splint wearing. Negative reactions to medications were noted by 43% of parents, with the most common reactions being complaining about taking medications, forgetting to take them, and refusing to take them. With reference to exercises, 60% of parents reported that their children had negative reactions, with the most frequent being complaining, refusing to do the exercises, and crying. Also, 43% of parents reported that their children had negative reactions to wearing splints; the most common reactions were refusing to wear splints, questioning the efficacy of splints, and being embarrassed about wearing splints at school or around friends. In the second study [13], an adherence questionnaire was administered to 93 parents of children with JRA and to 41 of the children with JRA. Adherence was also reported to be lower for exercises as compared to medications. The proportion of parents and children reporting adequate adherence to medications (95% and 89%, respectively) was significantly greater than the proportion of parents and children who reported adequate adherence to exercises (67% and 47%, respectively). In the third study [14], fifty patients with juvenile idiopathic arthritis and their parents completed questionnaires asking them about adherence to medications, exercises, and splints. Specifically, they were asked about the frequency at which children followed the treatments, difficulties in following the treatments, any negative reactions to treatments, and the degree to which the treatments actually helped. Both parents and children rated adherence to medications as higher than adherence to exercises (means of 84.9% and 83.1% for medications versus means of 61.2% and 57.4% for exercises, for children and parents respectively). Compared to their children, parents rated them as having more difficulty doing exercises and as having more negative reactions to take medications and doing exercises.

In the aggregate, these data suggest that the extent of adherence to treatments for JRA can vary widely across different samples and methods of assessing adherence but appears to be similar to what has been found with other chronic pediatric diseases [2]. The three survey studies would suggest that adherence to therapeutic exercises for JRA is more problematic than adherence to medications. What is not known is the optimal or minimal level of adherence necessary to produce acceptable disease and quality of life outcomes for patients. Clearly, there are some patients with JRA who may benefit from efforts to their enhance adherence. We now examine studies which have attempted to enhance adherence to regimens for JRA.

### Research on improving adherence to regimens for JRA

Two studies by our group have examined the efficacy of parent-managed token reinforcement programs in altering adherence to regimens for JRA. These behavior modification programs involve awarding tokens (points or poker chips) to children for adhering to regimen components, taking away tokens for nonadherence, and allowing children to purchase special or routine privileges with tokens. The first study [8] focused on improving adherence to medications, splint wearing, and prone lying (to prevent hip contractures) for a 7-year-old female with severe systemic-onset JRA. Adherence was assessed by parental observations with acceptable interobserver reliability (94%) obtained with an investigator conducting independent observations in the home. A multiple-baseline-across-behaviors design was employed to evaluate the effects of the intervention on adherence. Mean baseline adherence was low for medications (59%) and nil (0%) for both splint wearing and prone lying. Introduction of the token system increased adherence to 95% for medications, 77% for splint wearing, and 71% for prone lying. At the 10-week follow-up (with the token system withdrawn), adherence to medications, splint wearing, and prone lying averaged 90%, 91%, and 80%, respectively. Although not formally assessed, the pediatric rheumatologist anecdotally noted concomitant improvements in function for this patient, such as greater hip extension.

The second study [9] also tested the efficacy of a token system program in improving adherence to medications for a 14-year-old male with polyarticular JRA. Adherence was assessed by weekly pill counts obtained from the patient's mother over the phone with independent counts by an investigator in the clinic (agreement with the mother's count was 100%). A withdrawal (reversal), single-subject design was employed to evaluate the effects of the intervention on adherence and several clinical outcome parameters (e.g., active joint counts). Medication adherence averaged 44% during baseline, increased to an average of 59% during a simplified regimen condition (when the dosage was reduced from four to three times a day), and further increased and remained at 100% during the first token system phase. There was a decreasing trend in adherence during a token system withdrawal phase (mean = 77%), an increase during the second token system phase (mean = 99%), and an average of 92% during the maintenance phase (when the token system was not in effect but could be reinstated if adherence dropped below 80% for two consecutive weeks). At the nine-month follow-up (no token system in effect and no contingency for reinstatement), adherence averaged 97%. Though not as straightforward as the adherence results, there were improvements in clinical outcomes during the token system and follow-up phases (e.g., 5 active joints) relative to baseline and the token system withdrawal phase (e.g., 10 active joints).

Although the above-mentioned studies showed that token systems could be effective in improving adherence, they are labor-intensive for families and require well-trained personnel to implement and monitor. Therefore, our group reported on another study [10] that evaluated less complex behavioral strategies (such as self-monitoring of adherence by children and parents and positive verbal feedback by parents for adherence) combined with educational strategies (verbal and written information about medications for JRA, the importance of adherence, and strategies for improving adherence). The study involved three female patients with JRA, aged 3, 10, and 13 years and adherence was assessed by weekly pill counts obtained from parents over the phone with independent counts by one of the investigators in the clinic (agreement was 100%). A multiple baseline across subjects design was used to evaluate the efficacy of the intervention. Baseline medication adherence averaged 38% and 54% for two of the patients and increased during the intervention phase to an average of 97% and 92%, respectively. Adherence only increased slightly for the third patient, from an average of 44% during baseline to an average of 49% during the intervention phase. Adherence decreased for all three patients at four-month follow-up (means ranged from 24% to 89%). Interestingly, the patient for whom the intervention was least effective was a 13-year-old who had less parental supervision of her regimen and whose mother admitted she was nonadherent to medications prescribed to treat her arthritis. Unfortunately, clinical outcomes were not reported for these patients.

The above-mentioned studies suggest that adherence can be improved by behavioral strategies alone or combined with educational strategies, a combination that has worked best for improving adherence to other chronic pediatric diseases [2]. However, these studies involved small sample sizes and did not generally assess clinical outcomes, such as active joint counts. These studies also involved patients who had been diagnosed with having JRA for varying lengths of time and for whom nonadherence was implicated as interfering with the effectiveness of treatments by their pediatric rheumatologist. The success of these interventions with limited numbers of patients who were persistently nonadherent led our group to test the possible benefits of intervening with newly diagnosed patients in order to prevent the anticipated drop in adherence over time which has been reported in the pediatric adherence literature [2].

We conducted a randomized controlled trial evaluating a clinic-based, nurse-administered educational and behavioral intervention to promote adherence to nonsteroidal medications among newly diagnosed patients with JRA [15]. Thirty-four participants (mean age = 8.44 years) were matched by age and type of JRA and then randomly assigned to the experimental or (attention-placebo) control groups. Patients and parents in the experimental group were given verbal, written, and audiovisual information from a nurse about adherence improvement strategies, including prompting, monitoring, positive reinforcement, and discipline techniques [16]. Control group patients and parents were given verbal, written, and audiovisual information about JRA and treatments by the same nurse, but no specific information about adherence improvement strategies. Patients and parents in both groups received their respective interventions during a 11/2 hour clinic visit and were then telephoned by the nurse biweekly for two months and then monthly for 10 months. The content of the phone calls centered on the information presented during the initial clinic visit.

Adherence was assessed using the Medication Event Monitoring System (MEMS; Aprex Corporation, Fremont, CA). This electronic medication bottle cap records the date and time of each bottle opening. It can store 1800 openings and has an 18-month battery life. MEMS data are downloaded to a portable computer and analyzed with the manufacturer's software, which provides daily and continuous data on adherence (assuming that medications are taken each time the pill cap is removed). The daily MEMS adherence score was the percent of prescribed doses taken within the recommended dosing interval, with a two- hour (plus or minus) forgiveness interval (e.g., NSAID twice daily, could be taken 10 to 14 hours between doses). Disease activity and functional status measures were obtained during routine clinic visits where the rheumatologist recorded standard clinical indices, including number of active joints (those with pain, swelling, and limitation of motion), the number of minutes of morning stiffness, and a global disease activity rating (0 = off medication, in remission, 1 = quiescent, 2 = mild, 3 = moderate, 4 = severe). During these visits, the parents completed the Childhood Health Assessment Questionnaire (CHAQ), which is designed to assess disease-related functional limitations over the past week in eight areas: dressing and grooming, arising, eating, walking, hygiene, reach, grip, and play. The eight scores are averaged to yield the CHAQ Disability Index, ranging from 0 (no difficulty) to 3 (unable to do).

For the 52-week post-intervention follow-up, the experimental-group participants showed significantly better overall average adherence than the controls (77.7% vs. 56.9%, *p *= .023) and as predicted, the trend in adherence levels significantly dropped over time in the control group but not in the experimental group. There were, however, no significant post-intervention group differences on disease activity and functional status measures. The lack of significant differences in disease-related outcomes may have been due to "floor effects" (e.g., 68% of experimental participants and 67% of controls had quiescent or mild disease at baseline). This floor effect may have prevented detection of improvements that could be unambiguously attributed to the experimental adherence intervention.

A recent and unique randomized clinical trial focused on preventing osteoporosis in children with JRA by increasing calcium (Ca) intake [17]. Forty-nine children with JRA (mean age 6 years) and their parents were randomly assigned to a behavioral intervention (BI) group or an enhanced standard of care (ESC) group. Children and parents in the BI group met in separate groups for six sessions and received nutritional counseling on how to increase calcium intake and behavioral strategies (praise coupled with use of a sticker chart to track progress for reaching targeted calcium intake levels). Children and parent in the ESC group were seen individually for 3 visits and received nutritional counseling only. Three-day food diaries were kept by parents at baseline and posttreatment and were analyzed for calcium intake. Repeated measures analysis demonstrated a significant group by time interaction with children in the BI group achieving a greater increase in dietary Ca intake from baseline to posttreatment compared to the ESC group (*p *< .001). In addition, and of clinical significance, 92% of children in the BI group achieved the treatment goal of 1500 mg of Ca/day compared to 17% of children in the ESC group at posttreatment.

The above mentioned studies suggest that behavioral strategies combined with education is the most effective way to improve adherence to regimens for JRA and to prevent deterioration in adherence over time in newly diagnosed patients. The one dietary study also suggests that behavioral strategies are a necessary adjunct to nutritional education in improving calcium intake for patients with JRA. These findings are consistent with adherence intervention studies for other chronic pediatric diseases such as asthma, cystic fibrosis, and diabetes [2]. There are, however, too few adherence intervention studies and the ones which have been published involve small sample sizes, utilize less objective measures of adherence such as pill counts, and often fail to demonstrate that improvements in adherence produce improvements in disease and quality of life outcomes.

### Enhancing adherence in clinical practice

By combining the results of pediatric rheumatology adherence intervention studies with a larger database in pediatric adherence research [2], strategies for enhancing adherence can be recommended for clinical use. Studies that have been done suggest a three-tiered approach to minimize nonadherence: primary, secondary, and tertiary prevention [18]. *Primary prevention *efforts would be most relevant for those patients who have not yet exhibited clinically significant nonadherence (inconsistencies in following a particular regimen that may result in compromised health and well-being); possibly those recently diagnosed or those who are able to sustain adequate adherence over time. Interventions at this level would involve educational (e.g., stressing the importance of adherence), organizational (e.g., simplifying regimens), and relatively simple behavioral strategies (e.g., monitoring of regimen adherence by providers or parents). *Secondary prevention *might be most applicable to those patients for whom clinically significant nonadherence has been identified early on in the disease course or has yet to compromise their health and well-being. Interventions at this level might include more frequent monitoring of regimen adherence by parents and patients, specific and consistent positive social reinforcement for adherence, and general discipline strategies (e.g., time-out for younger children). Pediatric psychologists could train primary health care providers, particularly nurses, to implement primary and secondary level interventions. *Tertiary prevention *efforts would apply to patients with an ongoing pattern of clinically significant nonadherence. Strategies at this level might include token system programs, contingency contracting, self-management training (e.g., problem-solving to anticipate and manage obstacles to adherence), and possibly psychotherapy. Because of the demanding and technical nature of these strategies, pediatric psychologists would be responsible for implementing strategies at this level.

Implementing and evaluating primary, secondary, and tertiary prevention approaches to medical nonadherence depends on a number of factors. First, prevention efforts require a valid, reliable, and clinically feasible way to detect or assess nonadherence. Although no such "ideal" measure exists, 24-hour recall interviews (in clinics or by phone) may be the best option in that they have been shown to be reliable, valid, and feasible for routine and serial assessments of adherence to regimens for diabetes and cystic fibrosis and could be easily be adapted for JRA regimens [2]. Second, information obtained from routine and serial assessments of adherence should also allow for the detection of clinically significant nonadherence. Previous attempts at determining levels of adherence necessary to prevent deleterious health outcomes have been arbitrary and not biologically-based (e.g., adequate adherence defined as consuming 80% of prescribed medication doses). Third, because the desired outcome of adherence interventions is that patients get better, feel better, and do better, there is a need for both traditional (e.g., clinical signs and symptoms) and quality-of-life measures of disease and health status that are valid, reliable, and clinically feasible [2]. In conjunction with disease and health status measures, serial assessments of adherence would help determine the optimal or minimal level of adherence necessary to produce acceptable treatment outcomes. Fourth, interventions must take into account the age and developmental status of children. Interventions with younger patients would necessarily involve extensive parent involvement, while those with adolescents would focus on engaging them in problem-solving and other methods of enhancing their own adherence. Finally, because JRA affects relatively small numbers of children and adolescents, empirical validation of primary, secondary, and tertiary prevention interventions will require multi-center collaborative research studies.

### Improving adherence from a rheumatologists' viewpoint

Pediatric rheumatologists need to discuss adherence issues with their patients. This is evident from one chart review study that examined the medical records of 108 patients with JRA followed in a pediatric rheumatology clinic over a 15 month period [19]. Of the 2,578 treatment recommendations recorded in the charts, only 1,390 adherence-facilitating behaviors were documented, with the most common one being that staff informed the patient how often to perform treatments. The providers rarely indicated that they addressed patients' concerns or barriers relating to implementing treatments and they rarely recommended behavior modification strategies.

Pediatric rheumatologists can keep abreast of the adherence improvement literature but need practical advice on how to improve adherence based on the best evidence available and their own clinical experience. Table 1 provides just such a list of recommendations for enhancing adherence in clinical practice. If these recommendations are routinely implemented in clinical practice, most adherence problems can be circumvented or addressed in a timely fashion.

**Table 1:** 

Recommendations for Enhancing Adherence in Pediatric Rheumatology
1. Educate patients and families about the goals of treatment. Negotiate with them upfront about which treatments they are willing to try.
2. Make regimens as simple as possible and consistent with the patients' daily routine.
3. Educate patients and families about how to minimize treatment side effects and problem solve with them to address other barriers to adherence.
4. Ensure that patients and families have the requisite behavioral skills to implement regimens. Rehearse these in the clinic (e.g., demonstrate and have patients practice therapeutic exercises).
5. Encourage patients and caregivers to monitor adherence (e.g., use a calendar posted in a prominent place in the home).
6. Teach caregivers positive reinforcement strategies for promoting adherence (e.g., point system for adhering to regimen components).
7. Review discipline strategies with caregivers for children who are oppositional (e.g., time-out for younger child who refuses medications).
8. Teach older patients self-management strategies (e.g., problem-solving).
9. Refer patients and families to qualified mental health providers if more serious problems exist concurrently with nonadherence or are directly interfering with adherence.

## Competing interests

The author(s) declare that they have no competing interests.

## Authors' contributions

Manuscript Preparation: Rapoff and Lindsley
